# A Reverse Transcriptase-PCR Assay for Detecting Filarial Infective Larvae in Mosquitoes

**DOI:** 10.1371/journal.pntd.0000251

**Published:** 2008-06-18

**Authors:** Sandra J. Laney, Caitlin J. Buttaro, Sabato Visconti, Nils Pilotte, Reda M. R. Ramzy, Gary J. Weil, Steven A. Williams

**Affiliations:** 1 Department of Biological Sciences, Smith College, Northampton, Massachusetts, United States of America; 2 Amherst College, Amherst, Massachusetts, United States of America; 3 National Nutrition Institute, Cairo, Egypt; 4 Infectious Disease Division, Department of Internal Medicine, Washington University School of Medicine, St. Louis, Missouri, United States of America; 5 Program in Molecular and Cellular Biology, University of Massachusetts, Amherst, Massachusetts, United States of America; University of South Florida, United States of America

## Abstract

**Background:**

Existing molecular assays for filarial parasite DNA in mosquitoes cannot distinguish between infected mosquitoes that contain any stage of the parasite and infective mosquitoes that harbor third stage larvae (L3) capable of establishing new infections in humans. We now report development of a molecular L3-detection assay for *Brugia malayi* in vectors based on RT-PCR detection of an L3-activated gene transcript.

**Methodology/Principal Findings:**

Candidate genes identified by bioinformatics analysis of EST datasets across the *B. malayi* life cycle were initially screened by PCR using cDNA libraries as templates. Stage-specificity was confirmed using RNA isolated from infected mosquitoes. Mosquitoes were collected daily for 14 days after feeding on microfilaremic cat blood. RT-PCR was performed with primer sets that were specific for individual candidate genes. Many promising candidates with strong expression in the L3 stage were excluded because of low-level transcription in less mature larvae. One transcript (TC8100, which encodes a particular form of collagen) was only detected in mosquitoes that contained L3 larvae. This assay detects a single L3 in a pool of 25 mosquitoes.

**Conclusions/Significance:**

This L3-activated gene transcript, combined with a control transcript (*tph-1*, accession # U80971) that is constitutively expressed by all vector-stage filarial larvae, can be used to detect filarial infectivity in pools of mosquito vectors. This general approach (detection of stage-specific gene transcripts from eukaryotic pathogens) may also be useful for detecting infective stages of other vector-borne parasites.

## Author Summary

The Global Programme for the Elimination of Lymphatic Filariasis (GPELF) was launched in the year 1998 with the goal of eliminating lymphatic filariasis by 2020. As the success of mass drug administration (MDA) in the global program drives the rates of infection in endemic populations to very low levels, the development of new, highly sensitive methods are required for monitoring transmission by screening mosquitoes for the presence of L3 infective larvae. The current method of mosquito dissection to identify L3 larvae is laborious and insensitive and is not amenable to screening large numbers of mosquitoes. Existing molecular assays for the detection of filarial parasite DNA in mosquitoes are sensitive and can easily screen large numbers of vectors. However, current PCR-based methods cannot distinguish between infected mosquitoes that contain any stage of the parasite and infective mosquitoes that harbor third stage larvae (L3) capable of establishing new infections in humans. This paper reports the first development of a molecular L3-detection assay for a filarial parasite in mosquitoes based on RT-PCR detection of an L3-activated gene transcript. This strategy of detecting stage-specific messenger RNA from filarial parasites may also prove useful for detecting infective stages of other vector-borne pathogens.

## Introduction

Lymphatic filariasis (LF) is a disabling tropical disease that is caused by filarial nematode parasites that are transmitted by mosquitoes. *Brugia malayi* and *B. timori* account for approximately 10% of the global LF burden of 120 million infected individuals [1]. The Global Programme to Eliminate Lymphatic Filariasis (GPELF) has the ambitious goal of eliminating this disease by the year 2020 [2]. The program is largely based on a strategy of mass drug administration (MDA) of antifilarial medications to endemic populations with the aim of reducing human infection rates to levels that cannot sustain transmission by mosquitoes.

Improved tools are needed for monitoring progress in LF elimination programs. Currently, there are diagnostic assays to measure antigenemia [3], microfilarial loads, and antifilarial antibodies in humans [4],[5]. PCR detection of filarial DNA in mosquito vectors, termed molecular xenomonitoring (MX), is an important tool for indirectly detecting the presence of filarial infections in the human population [6]–[12]. MX is highly sensitive and is less intrusive than human blood collection for monitoring filarial infections in communities [13],[14]. However, positive DNA detection in vectors does not necessarily indicate that transmission is ongoing in a community. This is because PCR assays detect any stage of the parasite present in the vector, not just the infective stage (L3). Fischer and colleagues have recently shown that filarial DNA can be detected by PCR in non-vector species for at least two weeks following the ingestion of infected blood [15]. Thus, while filarial DNA assays efficiently detect filarial parasites in communities, they may not accurately reflect transmission of new infections.

A specific molecular test for infective mosquitoes would be a useful tool for monitoring the success of LF elimination programs. Although other diagnostic tools are available to measure LF in communities, “the ultimate test of interruption of transmission rests on infectivity rates of the mosquito vectors” [16]. Until now, the only way to measure infectivity in mosquitoes was by the laborious method of mosquito dissection. Dissection is not practical for detecting and measuring mosquito infection and infectivity rates when rates are very low following MDA.

This paper describes a *B. malayi* mRNA transcript that is first expressed in mosquitoes by infective larvae (a “L3-activated transcript”) and the development of a RT-PCR assay based on this transcript to detect L3 in mosquitoes. Molecular L3 detection can be used to screen large numbers of mosquitoes. This new tool holds great promise as a means of monitoring LF elimination programs and for detecting resurgent transmission of filariasis following the completion of MDA programs.

## Methods

### Bioinformatics selection of gene candidates

The *B. malayi* EST database [17] has been assembled into clusters of homologous sequences that each represent one gene by The Institute for Genomic Research (TIGR) [18]. We searched the *B. malayi* Gene Index Database (http://compbio.dfci.harvard.edu/tgi/cgi-bin/tgi/gimain.plgudbb_malayi) for candidate L3-activated genes that might be useful as targets for an L3-detection assay in mosquitoes. Target genes were called ‘L3-activated’ because their expression did not have to be limited to the to L3s for them to be useful for detecting infective mosquitoes. “L3-activated” genes are first expressed in L3 larvae. Thus, expression in the L4 through mature adult stages (the mammalian host stages) would not exclude a gene from being a potential target for detecting L3 larvae in mosquitoes. Of course, genes that are expressed in less mature filarial larvae in mosquitoes (e.g., Mf, L1 or L2) would not be useful for this purpose. The primary search criterion used to select gene candidates from the database was expression in BmL3 or BmL4 stage cDNA libraries with no expression in pre-L3 stage libraries. Other gene targets were included for testing based on previous reports of larval specificity [19] or on previous bioinformatics analyses of the *B. malayi* Expressed Sequence Tag (EST) dataset [20]. Candidate genes were removed from the list of potential targets in the following situations: 1) a BlastN search of the dbEST database (http://www.ncbi.nlm.nih.gov/BLAST/) yielded orthologous genes from other filarial species that were expressed in mf, L1, or L2 stage parasites; or 2) when intron-exon boundaries could not be determined. Exon boundary identification was necessary for primer and probe design in order to prevent detection of corresponding genomic DNA (gDNA) sequences.

### PCR screening of candidate L3-activated genes using cDNA libraries

Intron-exon boundaries were identified in the genomic sequences obtained from the *B. malayi* Genome Project [21] (www.tigr.org) using the mRNA-to-genomic DNA alignment program, SPIDEY (www.ncbi.nlm.nih.gov/spidey). Specific primers were designed for each candidate gene with the Oligo 5.1 primer design program (National Biosciences, Plymouth, MN). One primer for each gene was designed to span an exon-exon boundary to reduce the possibility of amplifying contaminating genomic DNA in cDNA library preparations. PCR was performed with phage template (2.5 µl) from the available stage-specific cDNA libraries: BmMf, BmL2, and BmL3 (available at http://www.filariasiscenter.org/ which is supported by NIAID contract RR211-258/6582667) in a 25 µl reaction with PCR buffer (Applied Biosystems, Inc.), 600 nM dNTPs, 200 nM each forward and reverse primer, 1.5 mM magnesium chloride, and Taq Gold enzyme (Applied Biosystems, Foster City, CA). Cycling parameters were 94°C for 10 min, 55°C for 5 min, followed by 35 cycles of 72°C for 90 sec, 94°C for 45 sec, 55°C for 45 sec, and a 10 min extension at 72°C. PCR products were detected by agarose gel electrophoresis. Candidate genes were excluded from further testing if amplification products were detected in the BmMf or BmL2 cDNA libraries.

### Mosquito feeding on microfilaremic blood

Infected *Aedes aegypti* (black-eyed Liverpool strain, AeL) mosquitoes were provided by the Filariasis Research Reagent Repository Center (FR3) at the University of Georgia (http://www.filariasiscenter.org/para-center/division.htm). Mosquitoes had been fed on blood from microfilaremic cats using artificial feeders with a natural skin membrane. Microfilaria counts in cat blood ranged from 80 to 150 mf per 20 µl. Blood-fed mosquitoes were maintained in an insectary at 27°C with 80% humidity. Mosquitoes were collected each day for 14 days and immediately preserved in RNAlater (Ambion). This period covered the extrinsic incubation period for *B. malayi* in mosquitoes. The FR3 center also provided uninfected control mosquitoes (either unfed or after feeding on blood from uninfected cats).

### RNA extraction from mosquito pools

RNA was extracted from pools of 5–8 mosquitoes using a phenol/guanidine thiocyanate extraction procedure. Preliminary studies showed that this method provided a more consistent and higher yield and quality of RNA than extractions using column based methods (data not shown). Mosquitoes were removed from RNAlater, blotted on a kimwipe to remove excess salt, and processed in pools in 2 mL round-bottom microcentrifuge tubes. Not all mosquitoes that fed on the infected blood were expected to have taken up parasites or to have supported the parasite's development Therefore, 2 or 3 mosquitoes that had fed on infected blood were included in each pool to increase the probability of having parasites present in each mosquito pool. Five biological replicates of all time points were tested. TRI reagent (Ambion, Inc.), a monophasic solution of phenol and guanidine thiocyanate, and glycogen [50 ug/mL] were added to each pool of mosquitoes, along with a zinc-plated 4.5-mm steel ball bearing (BB) (Daisy Outdoor Products, Rogers, AR). The parafilm sealed tubes were vortexed horizontally on high speed for 30 minutes for tissue disruption. Manufacturer's instructions were followed for TRI-reagent RNA extraction (Ambion, Inc.). This included the use of 1-bromo-3-chloropropane for the separation of the homogenate into aqueous and organic phases. To facilitate the removal of proteoglycan and polysaccharide contaminants, the RNA was precipitated using isopropanol with the addition of a high salt solution (0.8 M sodium citrate and 1.2 M NaCl), washed with 75% ethanol, and resuspended in 1 mM sodium citrate, pH 6.4 (RNA Storage Solution, Ambion, Inc.). To reduce genomic DNA contamination, all samples were treated with “DNA-free” (Ambion, Inc.) as per the manufacturer's instructions. RNA samples were evaluated using a Nanodrop spectrophotometer (NanoDrop technologies, Wilmington, DE) for quantity and purity.

### Conventional RT-PCR testing of infected mosquitoes

Conventional RT-PCR and multiplex RT-PCR reactions were performed using the OneStep RT-PCR kit (Qiagen, Inc.) with buffer containing 2.5 mM magnesium chloride, 0.4 mM dNTPs, 600 nM of the forward and reverse primers, 1 µl enzyme mix and 1 µl RNA template in a 25 µl total volume. The thermal cycling conditions used were 50°C for 30 min, 95°C for 15 min, and 55°C for 5 min, followed by 40 cycles of 72°C for 90 sec, 94°C for 45 sec, 55°C for 45 sec, and a 10 min extension step at 72°C. PCR products were electrophoresed on a 3% agarose gel, detected by ethidium bromide staining and visualized with ultraviolet light. The primers used in the multiplex conventional RT-PCR for the control gene *tph-1* amplify a 153 bp fragment from all stages of the parasite (#1054 5′-AAGGACGGCAAGTAGTAAGGA-3′ and #1059 5′-AACAGTTCATTTCTTGTAGC-3′). The primers for the L3-activated gene TC8100 amplify a 120 bp fragment (#1213 5′-TTTATTCATTAAAAGGCTTAGCATCT-3′ and #1207 5′-CATTTTGTAATGATATTCATCTCGAA-3′).

### qRT-PCR testing of mosquito pools

The Taqman OneStep RT-PCR kit (Applied Biosystems, Inc., Foster City, CA) was used according to manufacturer's instructions except the total reaction volume was 25 µl instead of 50 µl. The Primer Express v2.0 program (Applied Biosystems, Inc.) was used to design primer/probe sets for real time qRT-PCR. A 6-Fam labeled minor groove binding (MGB) probe was used for detection of the L3-activated gene products while a Taqman probe (Applied Biosystems, Inc.) was used for detection of the control gene products (*tph-1*). Both probes were designed to span an exon-exon boundary in order to prevent detection of any amplification resulting from residual genomic DNA contamination in the purified RNA. The primer sequences used for amplification of Bm-TC8100 were #1442 Bm-TC8100.F 5′-CCTGGTTTAAGCGGACAGGA-3′, and #1443 Bm-TC8100.R 5′-GCTGGCATGTTACCTGGAAGA-3′. The MGB probe sequence (Applied Biosystems, Inc., Foster City, CA) used for detection of the PCR product was: BmTC8100-MGB-FAM (6FAM-AACACCTGGTCTACC-MGBNFQ). The sequences of the Bm-*tph-1* primers for qRT-PCR were #1252 BmWb-tph1.F 5′-GACCGATTTAAACAGTTGCAGTTC-3′ and #1251 BmWb-tph1.R 5′-CTACTACAGCTACTTGTCCCTCACCTT-3′ and the Taqman probe sequence was #1250 6FAM-ATCGGTGAGCGTATGGCCGAAGG-Tamra.

Primer optimization was done for each primer/probe combination according to the Applied Biosystems, Inc. standard protocol. 2.5 µl of RNA extract were used in each 25 µl reaction: 12.5 µl Taqman OneStep RT-PCR Master Mix (Applied Biosystems), 0.625 µl 40× Multiscribe/RNase inhibitor, and 240 nM probe. The optimal primer concentration determined for TC8100 was 900 nM of each primer and for *tph-1* it was 300 nM forward primer (#1252) and 900 nM reverse primer (#1251). The plate was run using the Absolute Quantification module of the Sequence Detection System Program v1.3 on the Applied Biosystems 7300 Real-Time PCR System; all biological replicate samples were run in duplicate or triplicate along with gDNA and negative controls. The cycling conditions were 50°C for 30 min, 95°C for 10 min, followed by 40 cycles of 95°C for 15 sec and 60°C for 1 min.

### Multiplex qRT-PCR and conventional RT-PCR

The same Bm-*tph-1* probe sequence (see above) was labeled with VIC instead of FAM (Applied Biosystems, Inc.) for use in the multiplex qRT-PCR. Primer and probe optimizations were also done for the multiplex reaction according to the Applied Biosystems, Inc. standard protocol. The optimal concentrations were determined to be 900 nM of both the forward and reverse TC8100 primers, and 60 nM of the forward *tph-1* primer (#1252) coupled with 600 nM of the reverse *tph-1* primer (#1251). Both probe concentrations were 250 nM and all reactions were done in a 25 µl total volume with the Taqman One-step RT-PCR Master mix, and the Multiscribe reverse transcriptase enzyme with RNase inhibitor (Applied Biosystems, Inc.). 2 µl of template RNA were added to each reaction and standard qRT-PCR cycling conditions (listed above) were used.

### Assay sensitivity

Sensitivity level was measured both by mixing infective mosquitoes with unfed mosquitoes and by mixing individual BmL3 larvae with unfed mosquitoes. For detecting infective mosquitoes, one mosquito presumed to be infective (14 days after feeding on infected blood) was added to a pool of uninfected mosquitoes with total pool sizes of 5–25 mosquitoes. Three biological replicates were tested to control for the possibility that the presumed infective mosquito might not contain L3 stage parasites. In addition, three pools of two presumed infective mosquitoes with 3 negative mosquitoes were tested as positive controls. The sensitivity of the L3 detection assay was also evaluated directly by adding different numbers (10, 20, 25, or 30) of uninfected mosquitoes to tubes containing a single BmL3 parasite. RNA extraction and multiplex qRT-PCR were performed as described above.

### Specificity of the multiplex qRT-PCR assay

Mosquitoes were fed on *Brugia pahangi*, *Wuchereria bancrofti*, or *Dirofilaria immitis* infected blood and maintained in an insectary under standard conditions for times required for development of L3. Mosquitoes were collected on day 11 PBM for *B. pahangi*, on day 16 for *D. immitis*, and on day 15 for *W. bancrofti* and preserved in RNAlater. RNA was extracted from 3 biological replicates for each mosquito/parasite combination from pools comprised of 5 infected and 5 uninfected mosquitoes. All samples were tested in triplicate using the multiplex qRT-PCR diagnostic assay described above.

### Species confirmation testing

*B. malayi* and *B. pahangi* (a closely related cat parasite) are coendemic in some areas. Since TC8100 transcripts were detected in both *B. malayi* and *B. pahangi*, a different assay was needed to distinguish between these species. We used conventional RT-PCR with primers for the *rbp-1* gene [Accession # X91064]. Primers (#1662 *Bp-rbp1*.F1 5′-GAC GTC AGC TTC GTG TT-3′ and #1623-*Bp-rbp1*.R1 5′-AAC ATT TGA AAC GGG AT-3′) were designed to exploit a single nucleotide difference between *B. pahangi* and *B. malayi*. They amplify a 102 base pair product in *B. pahangi*; they do not amplify a sequence in *B. malayi*. Conventional RT-PCR using the standard manufacturer's protocol of the Qiagen One-step RT-PCR kit was performed with 600 nM of each primer and 1 µl of template RNA. The cycling and detection conditions were as described for conventional RT-PCR above except for the annealing temperature (57°C).

## Results

### cDNA Library PCR

Table 1 lists the candidate L3-activated gene targets generated via bioinformatics analysis of the *B. malayi* Gene Index that met the criteria listed in the Methods section. These genes were subjected to additional testing to determine the stage of onset of gene expression. Table 1 shows expression profiles determined for each candidate gene based on PCR amplification from BmMf, BmL2, BmL3, and BmL4 cDNA libraries (no BmL1 cDNA library is available). Twenty-three of 27 target gene candidates tested by cDNA library PCR had transcripts expressed in pre-L3 larval stage cDNA libraries. These candidates were not studied further. One candidate gene was not amplified in the vector-derived BmL3 stage cDNA library and was dropped from further consideration. Three candidate genes (TC7917, TC7969, and TC8100) were selected for further analysis as diagnostic targets based on successful amplification in the L3 stage cDNA library with no amplification in the pre-L3 stage cDNA libraries.

**Table 1:** cDNA Library PCR Screening of Gene Candidates.

Gene Identifier	Putative Identification based on Protein similarity matches	BmMf cDNA Library	BmL2 cDNA Library	BmL3 cDNA Library	Mammalian stage cDNA Libraries: (BmL3d6 or BmL4)
TC7714	Cuticle collagen	+		+	+
TC7770	Glyoxalase II		+	+	+
TC7785	No match		+	+	+
TC7804	Collagen	+	+	+	+
TC7822	Calcium-dependent kinase		+	+	+
TC7825	Serpin		+	+	
TC7866	*ard-1*, putative reductase		+	+	+
TC7871	*cpi-2*, cystatin	+	+	+	
TC7873	*alt-1*, abundant larval transcript		+	+	+
TC7897	Ubiquinol	+	+	+	+
TC7899	Unknown	+		+	
TC7908	Myosin, essential light chain	+	+	+	+
TC7917	Pyruvate dehydrogenase			+	
TC7969	*alt-3*, abundant larval transcript			+	
TC7999	Triosephosphate isomerase	+	+	+	+
TC8010	*cpi-1*, cystatin		+	+	+
TC8015	Unknown	+	+	+	+
TC8021	Glycosyl transferase		+	+	
TC8023	Unknown	+		+	
TC8026	Unknown		+	+	+
TC8100	Collagen			+	+
TC8154	Facilitated glucose transporter	+		+	+
TC8221	*cpi-1* cystatin				+
TC8253	SNS-Rich protein	+	+	+	+
TC8257	Unknown	+	+	+	+
TC8325	Unknown		+	+	NT
TC8383	Unknown	+	+	+	NT

Gene Identifier = TIGR Cluster Number (TC) available at http://compbio.dfci.harvard.edu/tgi/cgi-bin/tgi/gimain.plgudbb_malayi.

+ = PCR product detected, blank = no PCR product detected, and NT = not tested.

*B. malayi* cDNA library abbreviations: BmMf = microfilarial stage, BmL2 = L2 stage, BmL3 = vector-derived L3 stage, BmL3d6 = L3 larvae collected 6 days post mammalian infection, BmL4 = mammalian derived L4 stage.

### RT-PCR of candidate transcripts using RNA from infected mosquitoes

High quality RNA was extracted from pools of mosquitoes preserved in RNAlater with 260/280 nm absorbance ratios between 1.9–2.1 as measured by spectrophotometry. The three selected gene candidates were tested using RNA isolated from infected mosquito pools collected daily for 14 days post feeding on microfilaremic blood. A collagen gene (TC8100) had an expression signal by conventional RT-PCR first seen in mosquitoes 9 days after an infected blood meal (data not shown). This corresponds to the time when *B. malayi* L3 usually first appear in AeL mosquitoes reared under standard laboratory conditions. In addition, Bm-TC8100 qRT-PCR results confirmed this expression timeline (Table 2). Therefore, this gene was considered the best target for an L3 detection assay because it was determined to be unambiguously L3-activated.

**Table 2:** qRT-PCR Detection of the TC8100 Expression Profile Across a Two-Week Mosquito Time Course.

Mosquito Timepoint (#dPBM)	Expected stage of parasite development	TC8100 Ct value for Each of 5 Biological Replicates Representing the Stage of Expression				
		(3∶5)*	(2∶8)*	(2∶8)*	(2∶8)*	(2∶8)*
0	Mf	-	-	no RNA	-	-
1	Mf	-	-	-	-	-
2	L1	-	-	-	-	-
3	L1/L2	-	-	-	-	-
4	L1/L2	-	no RNA	-	-	-
5	L2	-	-	neg *tph-1*		
6	L2/L3	-	38.03	-	-	-
7	L2/L3	-	-	-	-	-
8	L2/L3	-	-	-	-	-
9	L3	35.33	33.68	31.17	neg *tph-1*	34.6
10	L3	34.36	33.27	30.32	34.09	35.65
11	L3	34.18	33.25	30.86	33.55	neg *tph-1*
12	L3	32.62	32.97	31.09	34.74	35.06
13	L3	35.01	34.83	31.84	36.36	35.32
14	L3	33.70	34.29	33.43	37.81	35.56

# dPBM = number of days post blood meal (after mosquitoes were fed on infected blood).

*: ratio of infected mosquitoes to total pool size.

No RNA indicates a faulty RNA Extraction as detected by spectrophotometry.

Neg *tph-1* indicates no parasite RNA was extracted in that mosquito pool.

“-” indicates no TC8100 RNA was detected in that sample.

Ct value = cycle threshold value (product amplification detected to cross the threshold).

The expression profiles of the other two candidate genes eliminated them as diagnostic targets. Conventional RT-PCR results indicated that TC7917 was first expressed on day 7 PBM. However, qRT-PCR, a more sensitive technique, detected expression on day 5 PBM, prior to the emergence of L3's in the vector. TC7969 (*alt-3*) expression was consistently detected by conventional RT-PCR at day 6 PBM corresponding to the earliest time point that *B. malayi* L3 have been identified in the bodies of insectary-reared AeL mosquitoes [22]. However, since no more than 10% of day 6 PBM mosquitoes are expected to contain BmL3 stage parasites and since there were 2 or 3 infected mosquitoes per pool, the detection of *alt-3* on day 6 PBM is most likely due to expression in L2 stage parasites. We then focused on TC8100.

### Expression profile of BmTC8100 and *tph-1* as determined by the qRT-PCR multiplex assay

Table 2 shows the expression profile of BmTC8100 in 5 biological replicates of the infected mosquito time course. Each mosquito pool contained 2 or 3 infected mosquitoes that had been collected at daily intervals beginning immediately after ingestion of infected blood (day 0) and continuing for 14 days. Of the 75 sample pools processed, 73 contained RNA of sufficient quantity and quality for testing as determined by spectrophotometry. Cycle threshold (Ct) values for *tph-1*, the constitutively expressed control transcript, were in the range of 23.5 to 37.6 with a mean value of 29.4. Ct values for BmTC8100, the L3-activated transcript candidate, were in the range of 30.2 to 39.1 with a mean value of 34.1. Ninety-six percent of the mosquito pools contained parasite RNA as indicated by positive amplification of the *tph-1* control gene. Four of the 5 replicate time-course studies detected TC8100 expression beginning on day 9 or day 10 PBM. This corresponds to the peak time for appearance of L3. TC8100 expression was detected on day 6 PBM in one of the five mosquito pool time course sets. TC8100 expression was not detected in mosquitoes from days 7 or 8 PBM in this set, but it was present in pools collected on days 9 through 14 PBM. TC8100 expression was not detected in any mosquito pool collected prior to day 6 PBM. These results indicate that TC8100 expression was only detected when L3 would be expected to be in mosquitoes; this is good evidence that the TC8100 collagen gene is L3-activated.

### Assay sensitivity

We assessed sensitivity of the TC8100 / *tph-1* multiplex qRT-PCR assay for detecting one *B. malayi* L3 in pools of 10, 20, 25 or 30 uninfected mosquitoes, with 3 biological replicates for each sample. RNA extracts ranged in concentration from 130–1826 ng/µl (Table 3). All samples tested positive for *tph-1* indicating that parasite RNA was extracted in all cases with Ct values ranging from 29.57–31.77. All of the samples with 10 to 25 mosquitoes were also positive for the L3-activated transcript, TC8100, with Ct values in the range of 36.40–38.67. However, only one of three biological replicates containing one L3 in a pool of 30 mosquitoes was positive for the L3 transcript. These results indicate that the multiplex qRT-PCR BmL3 detection assay reliably detected a single *B. malayi* L3 in mosquito pools less than or equal to 25.

**Table 3:** Detection of one *B. malayi* L3 added to pools of uninfected mosquitoes.

# BmL3 worms in the pool	# mosquitoes in the pool	RNA concentration (ng/*µl*)	*tph-1* qRT-PCR Ct value*	TC8100 qRT-PCR Ct value*
2 d14 PBM	30	130	30.0	38.2
2 d14 PBM	30	756	29.0	37.9
1	10	279	30.4	37.1
1	10	707	29.6	37.3
1	10	571	30.3	37.7
1	20	798	30.2	37.6
1	20	1198	31.0	37.2
1	20	1187	31.2	37.3
1	25	1400	30.8	36.4
1	25	1387	30.0	37.6
1	25	1360	30.9	38.7
1	30	1180	31.3	38.5
1	30	871	31.8	-
1	30	1826	30.6	-

*: All samples were run in triplicate and average Ct values are shown.

Dashed lines indicate that the TC8100 RNA was not detected in the sample.

To assess the sensitivity of the assay when L3 are located inside mosquitoes, we tested pools comprised of one day 14 PBM mosquito with varying numbers of uninfected mosquitoes (4–24). Since not all day 14 PBM mosquitoes would be expected to contain L3, it was not surprising that 7 of these pools tested negative for both *tph-1* and TC8100 RNA (Table 4). The other 14 pools all tested positive for both *tph-1* and TC8100 transcripts indicating that they contained L3. Of the 3 biological replicates for each pool size, at least one tested positive for TC8100, indicating that the assay can detect a single infective mosquito in a pool of 25 mosquitoes. Thus, both assessments of sensitivity suggested a maximum pool size of 25 mosquitoes.

**Table 4:** Detection of a single infective mosquito in mosquito pools.

# infective mosquitoes in the pool	Total Pool Size	RNA concentration (ng/*µl*)	*tph-1* qRT-PCR Ct value*	TC8100 qRT-PCR Ct value*
1	5	143	28.9	34.5
1	5	158	-	-
1	5	185	27.6	34.1
1	8	303	-	-
1	8	480	28.8	35.1
1	8	363	-	-
1	10	348	29.0	35.1
1	10	399	27.4	33.8
1	10	354	29.3	35.7
1	12	318	27.8	34.3
1	12	335	29.3	35.8
1	12	198	-	-
1	15	402	-	-
1	15	562	29.0	35.8
1	15	323	30.0	35.5
1	20	642	27.1	34.6
1	20	833	28.7	35.9
1	20	552	-	-
1	25	894	-	-
1	25	799	28.9	35.7
1	25	905	28.0	33.8

*: All samples were run in triplicate and average Ct values are shown.

Dashed lines indicate samples where no *B. malayi* RNA was detected.

### Assay specificity

*D. immitis* infected mosquitoes tested negative by real-time qRT-PCR for both the *tph-1* and the TC8100 transcripts using the primers and probes reported in this paper (Table 5). *W. bancrofti* infected mosquitoes tested positive for the *tph-1* control transcript but not TC8100, the L3-detection transcript. Both of these transcripts were detected in *B. pahangi* infected mosquitoes. These results indicate that the TC8100 *Brugia* L3 detection assay is genus-specific but not species-specific.

**Table 5:** Specificity Testing of the *Brugia* L3 Detection Assay.

RNA Sample No.	# infective mosquitoes (time PBM)	Total Pool Size	RNA concentration (ng/ul)	*tph-1* Ct value*	TC8100 Ct value*
BmL3-03	5(d14)	30	1373	28.2	34.9
DiL3-01	5 (d16)	10	694	-	-
DiL3-02	5 (d16)	10	448	-	-
DiL3-03	5 (d16)	10	573	-	-
WbL3-01	5 (d15)	10	712	24.5	-
WbL3-02	5 (d15)	10	739	25.5	-
WbL3-03	5 (d15)	10	997	25.6	-
BpL3-01	5 (d11)	10	583	24.5	30.5
BpL3-02	5 (d11)	10	827	25.1	31.0
BpL3-03	5 (d11)	10	737	26.8	34.6

*: All samples were run in triplicate and average Ct values are shown.

*BmL3* = *B. malayi L3*, *DiL3* = *D. immitis L3*, *WbL3* = *W. bancrofti L3*, *BpL3* = *B. pahangi L3 tph-1* is a constitutive target that is transcribed in all stages of *Brugia and Wuchereria*.

TC8100 is an L3-activated transcript that is only present in *Brugia* L3.

PBM = days post blood meal.

Dashed lines indicate that the RNA target was not detected in the sample.

### Species confirmation testing

A 102 base pair *rbp-1* RT-PCR product was detected in all *B. pahangi* samples, while all *B. malayi* samples were negative for the *rbp-1* transcript (data not shown) using the primers reported in this paper. This shows that the *rbp-1* assay can be used to detect the samples containing *B. pahangi*.

## Discussion

Several technical advances were required for a molecular L3 detection assay including a simple method for preserving parasite RNA in mosquitoes, a method for efficiently extracting RNA from parasites in pools of mosquitoes, identification of an L3-activated gene, and a method to sensitively detect that gene's transcript. We found that parasite RNA could be efficiently extracted from infected mosquitoes preserved in RNAlater (Ambion, Inc.) using a simple BB-grinding technique and a phenol-based extraction procedure. We then identified TC8100 as a L3-activated transcript and developed methods for its detection by conventional RT-PCR and qRT-PCR.

The *B. malayi* EST genome project provided an important starting point in our search for an L3-activated transcript with information on genes expressed in several parasite stages essential for our initial search. A L3 assay required an authentic L3-activated transcript with no expression in the early larval stages present in mosquitoes (Mf, L1 or L2). We carefully evaluated a panel of candidate genes; only the collagen gene TC8100 was confirmed to be a truly L3-activated transcript.

Time course studies were performed with RNA isolated from infected mosquitoes rather than with parasites isolated at different times after mosquito feeding. This approach proved the practical point that the target parasite sequence could be detected in infected mosquitoes preserved in RNAlater. The development of *B. malayi* in mosquitoes is not completely synchronous. Testing multiple replicates of infected mosquitoes at daily intervals and using 2–3 infected mosquitoes in each pool permitted us to be certain that we were covering the entire developmental timeline of the parasites in mosquitoes. Previous studies have documented the developmental time course of *B. malayi* in AeL mosquitoes reared under the laboratory conditions used in this study [22]. Within 48 hours of a blood meal, the majority of ingested microfilariae exsheathe and reach the ‘sausage’ stage (L1). The first molt to the L2 stage takes place between days 3–6, and the second molt to the L3 stage begins as early as day 6 but peaks between days 9–11 PBM. Therefore, a transcript that is truly L3-activated would be expected to have no expression prior to day 6 PBM, with most samples beginning expression by day 9, 10 or 11 PBM.

The expression profile of TC8100 was consistent with the BmL3 developmental timeline; the majority of the samples tested showed expression beginning at day 9 PBM corresponding to the peak timing of the L3 molt. Only one sample showed expression of TC8100 at day 6 PBM, which is the earliest reported time of L3 development. In addition to being activated at the beginning of L3 development, it is important to note that a successful diagnostic target must also continue to be expressed throughout the lifespan of the L3 parasite so that a mosquito containing L3 of any age can be detected. Our data showed that TC8100 transcripts were present in early L3 and in later L3.

The constitutively expressed *B. malayi* gene *tph-1* complemented TC8100 because it provided a useful marker to indicate the presence (and successful extraction) of filarial RNA in mosquito samples. The *tph-1* primers also amplified a homologous target sequence in RNA from the closely related filarial species *W. bancrofti* but not in RNA from the more distantly related animal filarid *D. immitis*. Therefore, in settings where there is no overlap between *Brugia* and *Wuchereria* infections, the TC8100/*tph-1* multiplex assay could be used to evaluate infection and infectivity rates simultaneously.

One consideration for the implementation of any new diagnostic technique is the practicality of using it as a surveillance tool. The storage of vectors in RNAlater eliminates any major limitations regarding mosquito collection. The mosquitoes can be stored for one day at ambient temperature and for at least several months at −20°C. Any laboratory that is already set-up to perform PCR would be able to use the conventional RT-PCR assay with no additional equipment investment. For the real-time assay, the investment of a real-time PCR machine would be necessary, but the cost of such instrumentation is dropping to levels as low as $16,000, making it a reasonable investment option. At this time, the cost of the RNA extraction and RT-PCR is approximately $5 per pool of mosquitoes, or $0.22 per mosquito. If the reactions are run in duplicate, the cost per pool would be approximately $6.30, or $0.25 per mosquito. This compares to the current cost of $5.00 per pool for the xenomonitoring DNA assay. The throughput for the conventional assay for a single technician would be estimated at 2,000 mosquitoes per week (80 pools with 25 mosquitoes per pool), while the real-time assay throughput is estimated at 4,000 mosquitoes per week (160 pools of 25 mosquitoes). The advantage to the real-time assay includes both a higher throughput level (reduced labor cost), as well as a reduction in potential contamination due to the elimination of post-PCR product handling. The real-time assay is a more cost efficient method, and thus, would be the recommended transmission surveillance tool wherever possible anticipating that the cost will decrease as time goes on.

A potential limitation of this L3 detection assay is that it cannot differentiate between *B. malayi* and *B. pahangi* (a zoonotic parasite) in mosquitoes. However, the same limitation applies to the traditional means of detecting L3 in mosquitoes, namely dissection with microscopy [23]. Our results showed that the *rbp-1* assay is specific for *B. pahangi*. This test can be used to help clarify results obtained from mosquitoes collected in areas where the two *Brugia* species are co-endemic. Only positive samples would need to be tested in this way, and the number of positive mosquito pools should become very small as infection rates fall in humans and mosquitoes following MDA.

One key limitation of this study is that our assays have not yet been tested with field-caught mosquitoes. Nevertheless, there have been many calls for the development of molecular tests for detection of filarial L3 in vectors [16],[24]. Our results serve as a proof of principle that L3-specific assays are feasible. Field studies are now needed to assess the practical value of such tests as tools for documenting the interruption of transmission in the context of filariasis elimination programs.

Finally, we need to note that the L3-detection assay based on TC8100 is specific to *Brugia* and does not detect L3 of *W. bancrofti*, the parasite responsible for the majority of the global burden of LF. Clearly, there is a need for the development of a diagnostic tool for the detection of WbL3 infective vectors as well. Surprisingly, we have been unable to identify the orthologue of this *Brugia* L3 activated gene, BmTC8100, in *W. bancrofti*. We are actively testing potential targets that can be used to detect *W. bancrofti* L3 in mosquitoes.

## References

[1] Michael E, Bundy DA (1997). Global mapping of lymphatic filariasis.. Parasitol Today.

[2] Ottesen EA (2000). The global programme to eliminate lymphatic filariasis.. Trop Med Int Health.

[3] Weil GJ, Lammie PJ, Weiss N (1997). The ICT Filariasis Test: A rapid-format antigen test for diagnosis of bancroftian filariasis.. Parasitol Today.

[4] Lammie PJ, Weil G, Noordin R, Kaliraj P, Steel C (2004). Recombinant antigen-based antibody assays for the diagnosis and surveillance of lymphatic filariasis - a multicenter trial.. Filaria J.

[5] Abdul Rahman R, Hwen-Yee C, Noordin R (2007). Pan LF-ELISA using BmR1 and BmSXP recombinant antigens for detection of lymphatic filariasis.. Filaria J.

[6] Williams SA, Laney SJ, Bierwert LA, Saunders LJ, Boakye DA (2002). Development and standardization of a rapid, PCR-based method for the detection of *Wuchereria bancrofti* in mosquitoes, for xenomonitoring the human prevalence of bancroftian filariasis.. Ann Trop Med Parasitol.

[7] Rao RU, Atkinson LJ, Ramzy RM, Helmy H, Farid HA (2006). A real-time PCR-based assay for detection of *Wuchereria bancrofti* DNA in blood and mosquitoes.. Am J Trop Med Hyg.

[8] Plichart C, Sechan Y, Davies N, Legrand AM (2006). PCR and dissection as tools to monitor filarial infection of *Aedes polynesiensis* mosquitoes in French Polynesia.. Filaria J.

[9] Plichart C, Laney SJ, Sechan Y, Davies N, Legrand A-M (2007). Correction: PCR and dissection as tools to monitor filarial infections of *Aedes polynesiensis* mosquitoes in French Polynesia.. Filaria J.

[10] Fischer P, Wibowo H, Pischke S, Ruckert P, Liebau E (2002). PCR-based detection and identification of the filarial parasite *Brugia timori* from Alor Island, Indonesia.. Ann Trop Med Parasitol.

[11] Lizotte MR, Supali T, Partono F, Williams SA (1994). A polymerase chain reaction assay for the detection of Brugia malayi in blood.. Am J Trop Med Hyg.

[12] Hoti SL, Vasuki V, Lizotte MW, Patra KP, Ravi G (2001). Detection of *Brugia malayi* in laboratory and wild-caught Mansonioides mosquitoes (Diptera: Culicidae) using Hha I PCR assay.. Bull Entomol Res.

[13] Bockarie MJ (2007). Molecular xenomonitoring of lymphatic filariasis.. Am J Trop Med Hyg.

[14] Farid HA, Morsy ZS, Helmy H, Ramzy RM, El Setouhy M (2007). A critical appraisal of molecular xenomonitoring as a tool for assessing progress toward elimination of Lymphatic Filariasis.. Am J Trop Med Hyg.

[15] Fischer P, Erickson SM, Fischer K, Fuchs JF, Rao RU (2007). Persistence of *Brugia malayi* DNA in vector and non-vector mosquitoes: implications for xenomonitoring and transmission monitoring of lymphatic filariasis.. Am J Trop Med Hyg.

[16] Gyapong JO, Twum-Danso NA (2006). Editorial: Global elimination of lymphatic filariasis: fact or fantasy?. Trop Med Int Health.

[17] Williams SA, Lizotte-Waniewski MR, Foster J, Guiliano D, Daub J (2000). The filarial genome project: analysis of the nuclear, mitochondrial and endosymbiont genomes of *Brugia malayi*.. Int J Parasitol.

[18] Liang F, Holt I, Pertea G, Karamycheva S, Salzberg SL (2000). An optimized protocol for analysis of EST sequences.. Nucleic Acids Res.

[19] Gregory WF, Atmadja AK, Allen JE, Maizels RM (2000). The abundant larval transcript-1 and -2 genes of *Brugia malayi* encode stage-specific candidate vaccine antigens for filariasis.. Infect Immun.

[20] Williams S, Laney S (2002). Filarial Genomics: Gene Discovery and Gene Expression. The Filaria.

[21] Ghedin E, Wang S, Spiro D, Caler E, Zhao Q (2007). Draft genome of the filarial nematode parasite *Brugia malayi*.. Science.

[22] Ramachandran CP (1966). Biological aspects in the transmission of *Brugia malayi* by *Aedes aegypti* in the laboratory.. J Med Entomol.

[23] Mak JW, Mak JW, Yong HS (1985). The identification of infective larvae..

[24] World Health Organization (2004). Technical Advisory Group on the Global Elimination of Lymphatic Filariasis (TAG-ELF): report of the fifth meeting, Geneva, Switzerland, 3–6 February 2004.

